# United States *Guild Certified Feldenkrais Teachers*®: a survey of characteristics and practice patterns

**DOI:** 10.1186/1472-6882-14-217

**Published:** 2014-07-02

**Authors:** Patricia A Buchanan, Nicole L Nelsen, Simon Geletta

**Affiliations:** 1 Toledo, Ohio 43614, USA; 2 St. Anthony Regional Hospital, Carroll, Iowa 51401, USA; 3 Master of Public Health Program, Des Moines University, 3200 Grand Avenue, Des Moines, Iowa 50312, USA

**Keywords:** *Feldenkrais Method* of somatic education, US *Guild Certified Feldenkrais Teachers*, Practitioner characteristics, Practice patterns, Communication, Decision-making, Research development

## Abstract

**Background:**

*Feldenkrais Method®* teachers help students improve function and quality of life through verbally and manually guided lessons. The reasons people seek *Feldenkrais®* lessons are poorly understood. Similarly, little is known about practice characteristics and patterns. To address these knowledge gaps, we conducted an extensive survey of United States *Guild Certified Feldenkrais Teachers®.*

**Methods:**

We invited all *Feldenkrais Teachers* to participate in this survey delivered in web-based or print formats. We obtained overall and question-specific response rates, descriptive statistics, chi-square tests of response bias, and performed qualitative thematic review of comments.

**Results:**

Overall response rate was 30.5% (392/1287). Ninety percent of responders had college degrees in diverse fields; 12.5% had credentials outside health care, 36.9% held conventional health care licenses, and 23.1% had complementary and alternative medicine credentials. Mean age was 55.7 years; most teachers were women (83%). California (n = 100) and New York (n = 34) had the most teachers. Forty-five percent of teachers earned ≤ 20% of their gross income from their practices, while 26% earned > 80%. Most saw < 10 students/week for individual lessons and < 10 students/week for group lessons. Students were mostly women (71.1%) and 45–64 years old. The primary reason students sought *Feldenkrais* lessons was pain. A quarter of students self-referred, a fifth were referred by conventional health care providers, and two-thirds paid for services directly. Themes from comments included: beliefs that *Feldenkrais* training had important personal and professional benefits for teachers; recognition of the challenges of operating small businesses and succinctly describing the *Feldenkrais Method*; the variety of practice approaches; and a deep commitment to the *Feldenkrais Method*.

**Conclusions:**

Most *Feldenkrais Teachers* were well educated, often held additional credentials, were located in the West, were women, were older than 50 years, and had part-time practices. Most students were women, were adults, came from various referral sources, and paid directly for services. Teachers and students utilized the *Feldenkrais Method* in diverse settings and applications. These findings may foster practice development by *Feldenkrais Teachers*, improve communication between health care consumers and providers and assist decision-making, and stimulate more research concerning the *Feldenkrais Method*.

## Background

An estimated half of the United States (US) population has used some form of complementary and alternative medicine (CAM) [1]. In 2010, US health care consumers spent $31 billion on direct payments to complementary and alternative medicine (CAM) providers [2]. Among these providers are certified teachers of the *Feldenkrais Method®* of somatic education. These teachers, practicing in more than 30 countries, work to improve the functions and quality of their students’ lives [3]. Through the use of verbally guided *Awareness Through Movement®* (ATM) lessons and manually facilitated *Functional Integration®* (FI) lessons, teachers (also known as practitioners) assist their students (also known as clients; typically not patients) to become more familiar with their current habitual behaviors, explore other movement options, and attend to their actions, sensations, emotions and thoughts [4,5]. These guided experiences presumably facilitate the emergence of more effective, flexible and adaptable behaviors [4,5].

International training standards guide the educational process to become a certified *Feldenkrais Teacher*. The curricula of *Feldenkrais* Professional Training Programs involve at least 800 hours distributed over 3–4 years to allow for considerable experience with the extensive repertoire of lessons, the development of teaching skills, and interdisciplinary study [6,7]. Teachers with additional credentials as Certified *Feldenkrais* Assistant Trainers and Certified *Feldenkrais* Trainers instruct participants in these programs. Only Certified *Feldenkrais* Trainers can serve as educational directors of training programs [6,7]. According to personal accounts, teachers enter training programs with a wide variety of backgrounds, credentials, and experiences. However, little research exists to support these reports.

The reasons people seek *Feldenkrais* lessons are poorly understood. Many individuals purportedly choose this method to aid with recovery from injury, manage chronic conditions, or enhance performance even though limited research supporting its safety and effectiveness exists to guide decisions about use and referral. Beyond anecdotal accounts, review of the literature suggests a broad range of concerns lead students to try *Feldenkrais* lessons. A small sample of reasons includes improvement of balance [8-10], problems with pain [11,12], chronic low back dysfunction [13,14], multiple sclerosis [15-17], various musculoskeletal conditions [18,19], and post-stroke functional limitations [20,21].

Similarly, little is objectively known about practice characteristics and patterns, such as practice volume, settings, and methods of payment. Present data about practitioners are mostly limited to information contained in the *Feldenkrais Guild* of North America (FGNA) membership print and on-line directories [22]. Entries specify year of certification, geographic location, and may include information about other credentials (e.g., PT, RN, MD) or advanced degrees, and practice specialties (e.g., performing arts, children, sports).

These knowledge gaps pose significant barriers to the assessment of safety and effectiveness by consumers, appropriate consideration of referrals by health care providers, and development of quality research studies. As an initial step in reducing these barriers, the first author conducted a small survey in 2009 to begin to collect and distribute information about the characteristics and practice patterns of US *Feldenkrais Teachers*[23]. Teachers were more commonly located in the West and Northeast. While most responders were singularly credentialed as *Feldenkrais Teachers*, nearly 23% were also physical therapists. Practice sizes varied considerably, but most teachers operated part time practices. Just over half provided exclusively *Feldenkrais Method* lessons in their sessions.

Many questions about provider characteristics and practice patterns went unanswered in that short survey and new questions arose from the results. Therefore, in 2011 we took the next step and conducted this more extensive survey of United States *Guild Certified Feldenkrais Teachers.* The purpose of this study was to gather in-depth information about *Feldenkrais Teachers* that would assist them with practice improvement, enhance communication between and decision-making by health care providers and consumers, and support development of relevant research. In addition to *Feldenkrais Teachers’* demographics, we gathered information about: credentials and education in the *Feldenkrais Method*, other CAM practices, and conventional health care; practice settings; and practice patterns.

## Methods

### Survey development

We used a survey to obtain a snapshot of the characteristics and practice patterns of certified *Feldenkrais Teachers* within the US. This survey was modeled after studies of other CAM providers by Cherkin and colleagues [24-28], adapted to the *Feldenkrais* field, and informed by the preliminary study [23]. The Des Moines University Institutional Review Board approved the study protocol and consent forms.

After drafting the survey, we sought feedback from 2 focus groups of US *Feldenkrais Teachers*. The first review occurred with 9 attendees at the 2010 FGNA Midwest regional meeting. We revised the survey in response to comments and obtained another review from 7 attendees of the 2010 *Feldenkrais Method* Annual Conference. We finalized the survey for delivery in either a web-based, secure format utilizing Feedback Server® 4 or in print for mail delivery.

The survey had 7 parts: 1) *Feldenkrais Method* training and certification, 2) educational background, 3) other licensures, certification, and specialties, 4) demographics, 5) overview of the past 12 months (January - December 2010), 6) review of representative month during the past 12 months (January - December 2010), and 7) open comment. There was a maximum of 108 questions, with skip logic included to direct responders away from questions that were not applicable to them based on previous responses. In the instructions, we encouraged participants to review their records, to take breaks as desired, and to review the definitions of terms (see Additional file 1) before beginning the survey. In the definitions of terms, we identified licensed or certified practitioners in health care fields that are recognized by the American Medical Association and similar groups operating within a conventional, Western medical model as traditional health care providers. Throughout this report, we use the current terminology of conventional health care (CHC) providers.

### Participants

We obtained the listing of all US *Guild Certified Feldenkrais Teachers* as of January 25, 2011 from FGNA. We mailed letters to everyone alerting them that they would soon receive an invitation to participate in the survey. The majority of *Feldenkrais Teachers* received the invitation, including consent information and a link to the survey, via email. Those who had a stated preference to be contacted by mail and those for whom email deliveries failed received the consent information and survey with postage-paid return envelopes via mail.

Teachers had a 2-month window (February 15 to April 14, 2011) to complete and submit the survey. To encourage participation, we sent up to 3 reminders by email or mail during this period. Persons who completed and returned the survey were eligible for 1 of 50 randomly-selected $50 payments to compensate participants for their time.

### Data processing and statistical analysis

Once the survey response window closed, we manually entered individual print survey responses into Feedback Server® 4. We exported data for all responders into Microsoft® Office Excel 2007 for screening and cleaning. Subsequently, we used IBM® SPSS® Statistics 19 to obtain question-specific response rates in the form of valid percentages (not all responders answered all questions), descriptive statistics, and chi-square tests of response bias. For our descriptive statistics, we determined mean ± standard deviation (SD) as measures of centrality and dispersion, and 95% confidence intervals to represent true values of key measures. We used information about *Feldenkrais Teachers* provided with the January 2011 listing, other *Feldenkrais* community resources, and internet searches to assess for geographic distribution, gender, and advanced *Feldenkrais* credential biases in the distribution of our responders. Finally, we exported responders’ comments to 3 open questions into Microsoft® Office Word 2007 for qualitative thematic review. Two authors used conventional, inductive open coding to independently review the comments, discussed their analyses, and finalized the themes. We selected exemplar quotations to illustrate the themes.

## Results

### Survey participation

**Figure 1:**
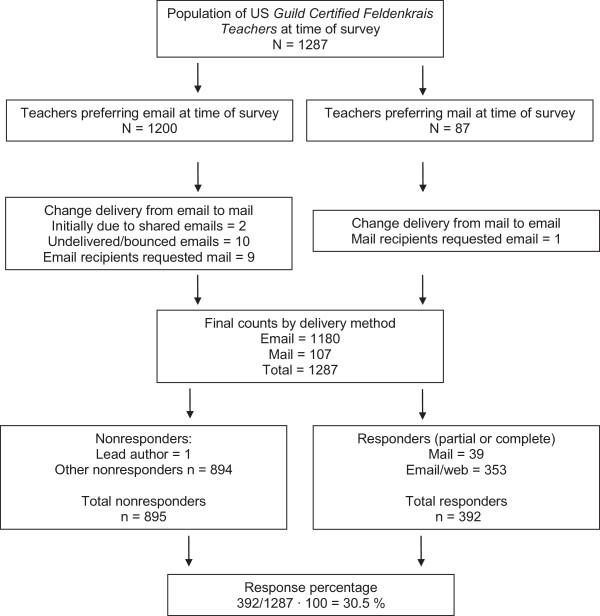
Population, delivery method counts, nonresponder and responder numbers, and survey response percentage.

Figure 1 summarizes the population, delivery method counts, nonresponder and responder numbers, and survey response percentage. The first author is a *Feldenkrais Teacher* and chose not to complete the survey.

### *Feldenkrais Method* training and certification

Nine of the responders (9/392 = 2.3%) graduated in 1977 from the first US *Feldenkrais* Professional Training Program held in San Francisco, California. Thirty-three of the responders (8.4%) graduated from training programs between 1981 and 1990, 179 (45.7%) between 1991 and 2000, and 171 (43.6%) between 2001 and 2010. For the 52 responders (13.3% of 392) who provided the year in which they first learned about or experienced the *Feldenkrais Method*, the time interval from the introductory year to graduation from a training program varied considerably (mean = 9.1 ± 7.9 years). In addition to certification in the *Feldenkrais Method*, 28.9% (113/391) of responders had additional certifications in 1 or more techniques that are substantially based on the teaching of Moshe Feldenkrais, DSc, originator of the *Feldenkrais Method*.

The majority of responders (361/392 = 92.1%) completed their training in the US, with programs held in 24 states and the District of Columbia. California had the most training program graduates with 156 (39.8%). Twenty of the responders (5.1%) completed training programs in Canada, 4 (1.0%) in multiple locations, and 7 (1.8%) outside of North America. Twenty-two different educational directors, solely or in partnerships, led the North American trainings. Moshe Feldenkrais led the trainings of 23 of the responders (5.9% of 392).

In this survey, 27 of the responders (6.9% of 391) were Certified *Feldenkrais* Assistant Trainers. Of the 26 who provided their year of credentialing, 6 (23.1%) became credentialed between 1981 and 1990, 14 (53.8%) between 1991 and 2000, and 6 (23.1%) between 2001 and 2010. Three responders reported they were in the process of applying to be Assistant Trainers.

Thirteen of the responders (3.3% of 391) were Certified *Feldenkrais* Trainers. Two (15.4%) became Trainers between 1981 and 1990, 7 (53.8%) between 1991 and 2000, and 4 (30.8%) between 2001 and 2010. One responder was in the process of applying to be a Trainer.

The 6.9% response rate of Assistant Trainers was more than double the 3.0% (38 of 1287) representation among the January 2011 listing. The 3.3% response rate of Trainers was higher than the 2.1% (27 of 1287) representation as of January 2011. The response rates of Assistant Trainers and Trainers were not statistically significantly different (*Χ*^*2*^ = 3.5, df = 1, P = 0.06). However, a significant chi-square test (*Χ*^*2*^ = 31.4, df = 1, P < 0.01) indicated there was a response bias with Assistant Trainers and Trainers more likely to respond than *Feldenkrais Teachers* without these advanced credentials.

### Educational background

All 388 responders (99.0% of 392) reported having at least a high school education. For highest degree earned, 47.7% (185 of 388) of the responders reported holding Bachelor’s degrees, 33.5% (n = 130) held Master’s degrees, and 9% (n = 35) held Doctorate degrees. In listing up to 5 degrees and majors, responders reported a highly diverse catalog of areas of study ranging from A (accounting) to Z (zoology). We grouped areas of study across all degrees into similar disciplines (e.g., zoology with biological sciences) and determined their frequencies. Physical therapy ranked first (n = 112) with more than twice the responses of any of the next most common fields of education (n = 44), music (n = 43), psychology (n = 38), and biological sciences (n = 35).

### Other licensures, certifications, and specialties

We first asked responders if they held current licenses or certifications that were not specific to conventional health care or CAM and, if so, to list them. Forty-eight of the 385 responders (12.5%) did. Most common were education licensures (n = 11) and exercise certifications (n = 5).

Next, we asked responders if they held current licenses or certifications as a conventional health care provider and, if so, to identify them. One hundred forty-one of 382 responders (36.9%) answered yes. Most of those responding yes (n = 133) held 1 conventional license, while 7 reported having 2 licenses. Among those credentials with more than 1 response, physical therapist was the clear leader (n = 93). There were responses from 15 occupational therapists, 5 nurses, 5 speech-language pathologists, 4 athletic trainers, 4 physical therapist assistants, 4 social workers, and 2 medical doctors.

Third, we asked responders if they were licensed or certified in CAM practices and, if so, to list them. Eighty-nine of 385 responders (23.1%) reported having 1 or more of these credentials. While most of those responding yes (n = 47) identified 1 CAM certification, 23 listed 2 certifications, 8 named 3 certifications, and 5 others reported 4–11 certifications. For certifications with more than 1 response, massage therapist was most common (n = 36). The subsequent certifications with more than 5 responses were Reiki (n = 13), Pilates (n = 12), tai chi (n = 9), yoga (n = 9), qigong (n = 7), reflexology (n = 7), craniosacral therapy (n = 7), and meditation (n = 6).

We considered what combinations of credentials (*Feldenkrais Method*, licenses or certifications not specific to conventional health care or CAM, conventional health care provider licenses, and CAM practice certifications) the 382 responders identified. While 40.3% were solely credentialed in the *Feldenkrais Method*, over half of responders were certified in 1 or more additional areas. The credentials combinations above 10% were *Feldenkrais Method* and conventional (27.7%), and *Feldenkrais Method* and CAM (13.1%).

We asked responders whether they promoted or advertised any of their nonspecific, conventional health care, or CAM provider credentials. Of the 215 (54.8% of 392) who answered, 25.6% promoted none, 55.3% promoted some, and 19.1% promoted all of their credentials. In addition, we asked responders if they had specialty areas that were not identified by these 3 groups of credentials. Ninety-two of 376 responders (24.5%) reported specialties. Of those, 90 clarified how they promoted their specialties: 21.1% promoted none, 46.7% promoted some, and 32.2% promoted all specialties. We categorized the reported specialties into related groupings and identified those with more than 3 responses. These 8 specialties were led by work with performing artists (n = 25), followed by applications with infants and children (n = 22), chronic pain (n = 15), elderly and aging (n = 11), neurological conditions (n = 9), sports, martial arts, yoga (n = 7), horses and riders (n = 6), and balance (n = 4).

Finally, we asked responders to think about all aspects of their practice and to identify the forms of promotion that applied to them. All teachers who were also members of FGNA had detailed listings in the FGNA print directory, could opt in or out of the FGNA online directory, and had an AltMed web listing they could develop. Non-members only had their names and years of certification listed in the print directory. The most to least frequently used forms of promotion for 25% or more of the 380 responders were the FGNA print directory (89.2%), FGNA online directory (87.6%), email (54.7%), flyers (50.3%), personal website (45.8%), and social media (25.0%). Among other forms of promotion (20.0%), word-of-mouth/personal referral (n = 16) was the most common response, followed by distribution of business cards (n = 5).

### Demographics

We asked teachers to identify their gender. Responders were 83.0% women (318 of 383) and 17.0% men. While we could not reasonably confirm the gender of 5 of 1287 teachers in the January 2011 listing, the response rates were similar to the list distribution of 81.9% women (1050 of 1282) and 18.1% men. Based on a non-significant chi-square test (*Χ*^*2*^ = 0.47, df = 1, P = 0.49), there was no indication of gender bias in the response sample compared to the population.

On average for 380 responders (96.9% of 392), teachers were 55.7 ± 9.2 years of age. There was 1 responder over the age of 79 and no responder younger than 29. Table 1 presents the distribution of age groups. The largest group of responders were in their 50s. The 27 Assistant Trainers averaged 57.9 ± 7.4 years of age, while the 13 Trainers were 62.2 ± 5.1 years old.

**Table 1:** **Profile of *Feldenkrais Teachers *** **and traditional format practice patterns during 2010**

**Characteristic**	**Responders**	**Percentage**	**Mean (SD)**	**95% CI**
Age group				
18–29 years	3	0.8		
30–39 years	14	3.7		
40–49 years	71	18.7		
50–59 years	153	40.3		
60–69 years	112	29.5		
70–84 years	27	7.1		
Region				
Northeast^a^	62	16.3		
Midwest^b^	50	13.1		
South^c^	65	17.1		
West^d^	204	53.5		
Gross income level				
Less than $25,000				
All	88	23.6		
Men	11	17.2		
Women	77	24.9		
$25,000 to $49,999				
All	124	33.2		
Men	21	32.8		
Women	103	33.3		
$50,000 to $74,999				
All	88	23.6		
Men	9	14.1		
Women	79	25.6		
$75,000 or more				
All	73	19.6		
Men	23	35.9		
Women	50	16.2		
Percentage of gross income from *Feldenkrais* practice				
0				
All	37	9.9		
Men	2	3.1		
Women	35	11.3		
1-20				
All	132	35.4		
Men	29	45.3		
Women	103	33.3		
21-40				
All	46	12.3		
Men	6	9.4		
Women	40	12.9		
41-60				
All	30	8.0		
Men	4	6.3		
Women	26	8.4		
Traditional practice format, 12-month overview questions				
How many weeks did you practice?	266	96.7	39.6 (14.3)	37.9-41.3
How many hours of direct contact with students did you have in a typical week?	265	96.4	10.5 (9.8)	9.3-11.7
How many appointments (visits) for individual lessons (*Functional Integration*® or *Awareness Through Movement*®) with students did you have in a typical week?	257	93.5	8.7 (8.6)	7.7-9.8
How many students did you have for individual lessons in a typical week?	255	92.7	7.9 (8.0)	6.9-8.9
How many minutes did your individual lessons last?				
Most often, typical lesson	260	94.5	58.4 (9.3)	57.3-59.5
Shorter lessons	174	63.3	43.5 (11.8)	41.7-45.2
Longer lessons	171	62.2	77.0 (14.9)	74.8-79.3
How many classes (visits) for group lessons (*Awareness Through Movement*) with students did you have in a typical week?	275	100.0	1.5 (2.4)	1.2-1.8
How many students did you have for group lessons in a typical week?	275	100.0	8.1 (11.2)	6.8-9.4
How many minutes did your group lessons (classes) last?				
Most often, typical lesson	190	69.1	58.7 (9.1)	57.4-60.0
Shorter lessons	102	37.1	48.7 (11.3)	46.5-51.0
Longer lessons	101	36.7	71.7 (23.5)	67.0-76.3
How many public workshops based on the *Feldenkrais Method* did you offer?	275	100.0	2.0 (3.2)	1.7-2.4
How many workshops for *Feldenkrais* practitioners and/or students in accredited *Feldenkrais* Professional Training Programs did you offer?	275	100.0	0.6 (2.4)	0.3-0.9

Item-specific numbers of responders and percentages for all characteristics; means with standard deviations (SD), and 95% confidence intervals (CI) for 12-month overview of traditional practice format.

^a^New York, Pennsylvania, Connecticut, Maine, New Jersey, Massachusetts, New Hampshire, Vermont, Rhode Island.

^b^Illinois, Michigan, Wisconsin, Indiana, Minnesota, Ohio, Iowa, Missouri, Kansas, North Dakota, Nebraska, South Dakota.

^c^Virginia, Maryland, North Carolina, Florida, Georgia, Texas, Alabama, Oklahoma, Louisiana, Tennessee, Delaware, South Carolina, West Virginia, Arkansas, District of Columbia, Kentucky, Mississippi.

^d^California, Washington, Oregon, Colorado, Arizona, New Mexico, Idaho, Hawaii, Utah, Alaska, Wyoming, Montana, Nevada.

Table 1 summarizes the numbers of responders (total = 381; 97.2% of 392) by geographical regions. Regional response rates were 16.3% for the Northeast (state with most responders: New York, n = 34), 13.1% for the Midwest (Illinois, n = 14), 17.1% for the South (Virginia, n = 15), and 53.5% for the West (California, n = 100). Geographical distribution based on the January 2011 listing (n = 1287) was 20.5% for the Northeast, 13.4% for the Midwest, 16.0% for the South, and 50.1% for the West. There was no indication of geographic bias among our responders per chi-square test (*Χ*^*2*^ = 6.41, df = 3, P = 0.09). There were no responders from 10 states (Northeast: Rhode Island; Midwest: Kansas, North Dakota, Nebraska, South Dakota; South: Arkansas, Kentucky, Mississippi; West: Montana, Nevada) and the District of Columbia. This compares with no teachers living in Delaware, North Dakota, and South Dakota as of the January 2011 listing.

We asked responders to identify their ethnicity and race. The majority of responders selected not Hispanic, Latino or Spanish as their ethnic background (98.4%) and identified their race as white (94.5%).

Responders provided information about their individual gross income from all sources during 2010. Table 1 indicates the frequencies and percentages for 4 levels of gross income. Women were more likely than men to have incomes under $25,000, while men were more likely than women to have incomes at or above $75,000. We next asked responders what percentage of their individual gross income during 2010 came from earnings related to their *Feldenkrais Method* practices. The mean percentage for the 373 responders (95.2% of 392) was 43.0% ± 39.3%. Grouping percentage of income from *Feldenkrais* practice into bins revealed a bimodal distribution (see Table 1) favoring the ranges of 1%-20% and 81%-100%. More women than men reported no income from *Feldenkrais* practice, yet more men than women reported 1%-20% income from *Feldenkrais* practice. Percentage of income differences between women and men were small for the other bins.

As another perspective on responders’ practices, we asked if they considered the practice of the *Feldenkrais Method* to be their primary occupation. Of the 377 responders (96.2% of 392), 46.7% marked yes; 53.3% selected no. We then asked responders to provide their primary occupation. Similar to the diversity of college majors, responders reported occupations from A (accounting) to Y (yoga). Of the 380 responders (96.9% of 392), the most commonly reported occupations with at least 10 responses were *Feldenkrais Teacher* (n = 40), physical therapist (n = 27), educator (n = 11), and retired (n = 10; n = 13 including semi-retired).

Finally, through a series of 3 questions, we screened responders for whether or not they practiced in a formal setting (meaning teachers were paid or compensated for their practice, and they worked with students other than family and friends) during 2010. When asked if teachers practiced the *Feldenkrais Method* in any manner at any time during 2010, 98.9% of responders (373 of 377) reported yes. Twenty of 368 responders (5.4%) reported they only practiced informally with family and friends, while 89.4% (330 of 369) reported practicing in a formal setting. We embedded skip logic in the survey to direct those who did not report having a formal practice to the last, open comment section.

### Overview of the past 12 months (January - December 2010)

We began this section by asking teachers what percentages of their *Feldenkrais Method* practices were in traditional and integrated formats (see Additional file 1 - Definition of terms). At this stage of the survey, 77.3% of the initial responders (303 of 392) answered that 68.8% ± 37.6% of their practices were in traditional formats and 31.2% ± 37.6% of their practices were in integrated formats.

Two hundred seventy-five of 305 responders specifically reported that part of their practices were in a traditional format. Those who responded yes answered several questions about their contacts with students, length of lessons, and delivery of workshops. Table 1 summarizes the question-specific numbers of responders, means, SDs, and 95% confidence intervals (CI) for these items.

One hundred sixty of 294 responders reported that part of their practices were in an integrated format. For 151 of those who responded yes, the mean weeks of practice was 37.4 ± 17.0 (95% CI 34.7 to 40.1). The mean hours of direct contact with students in a typical week of integrated practice for 147 of these responders was 13.9 ± 12.5 (95% CI 11.9 to 15.9).

We asked those who practiced in an integrated format to give a short description of how they integrated their practice of the *Feldenkrais Method* into their other occupation or professional context. Most of the 154 responders (39.3% of 392) basically stated with what they combined the *Feldenkrais Method*. As examples, “I integrate ATM lessons into voice therapy sessions,” and “When teaching yoga, I teach an ATM lesson around the theme of the class, i.e., twisting, reaching, sitting”. Many responders offered comments about the impact of a *Feldenkrais* perspective on their practice. One physical therapist stated, “I am a provider who respects the complexity of every individual and I do not impose force during my treatments”. Several commented on how deeply they have integrated the *Feldenkrais Method* into their work. One responder stated succinctly, “It is impossible to separate *Feldenkrais* [*Method*] from other modalities”. Another responder elaborated: “Since learning the *Feldenkrais Method*, I incorporate the process of looking at, feeling, finding origins of movement, feeling of pattern change into my speech/language work. I no longer tell a client what to do or how to do it based on a standard/model but ask him/her to find a way to make changes in current thinking/production outside level of comfort or habit in order to notice differences which may or may not become new and/or more useful patterns”. A final exemplar provides another perspective of the impact of the *Feldenkrais Method* on the teacher: “Awareness of self use from the *Feldenkrais Method* is a key point in my professional work”.

We asked responders to consider their whole practice, including traditional and integrated formats, in answering the remaining questions in this section. First, we inquired about what percentages of their practices involved students across 7 age groups. Most commonly, 297 responders (75.8% of 392) had students who were 45–64 years old (see Table 2). Second, we asked for the percentages of students who identified as female, male, or some other identity. Responders (295 of 392) reported that most students identified as females, with mean = 71.1% ± 17.6% (95% CI 69.1% to 73.1%). For male-identified students, mean = 28.8% ± 17.7% (95% CI 26.8% to 30.8%). Several responders worked with students who had some other identity, with mean = 0.1% ± 0.5% (95% CI 0.0% to 0.1%).

**Table 2:** **Percentages for age groups of students across responders’ whole *Feldenkrais Method *** **practices during 2010**

**Age group**	**Mean (SD)**	**95% CI**
0-4 years	6.2 (18.8)	4.0-8.3
5-11 years	3.9 (11.4)	2.6-5.2
12-17 years	2.9 (6.3)	2.2-3.7
18-24 years	5.1 (10.0)	3.9-6.2
25-44 years	18.0 (18.2)	15.9-20.1
45-64 years	40.4 (25.3)	37.5-43.3
65 years and above	23.5 (22.6)	20.9-26.1

Means with standard deviations (SD), and 95% confidence intervals (CI).

Next, we asked responders to identify the top 5 most frequent reasons that students came to their practices. We categorized all reasons into pain (7 subgroups) and non-pain (12 subgroups) groups (see Table 3). Pain was the most common reason students sought *Feldenkrais* lessons. Subsequently, non-pain reasons were more prevalent.

**Table 3:** **Top 5 most common reasons students sought *Feldenkrais *** **lessons during 2010**

**Reasons**	**Reason 1**	**Reason 2**	**Reason 3**	**Reason 4**	**Reason 5**
Main groups	n = 292	n = 289	n = 265	n = 216	n = 180
Pain	62.7	38.4	24.2	16.7	13.3
Non-pain	37.3	61.6	75.8	83.3	86.7
Pain subgroups	n = 183	n = 111	n = 64	n = 36	n = 24
General	73.8	45.9	42.2	38.9	20.8
Upper extremity	0.0	4.5	10.9	13.9	16.7
Lower extremity	0.5	3.6	9.4	22.2	16.7
Back	20.8	27.9	15.6	8.3	20.8
Spine, other	3.8	8.1	12.5	5.6	4.2
Upper quarter^a^	0.0	7.2	4.7	2.8	20.8
Lower quarter^b^	1.1	2.7	4.7	8.3	0.0
Non-pain subgroups	n = 106	n = 178	n = 201	n = 180	n = 156
Balance-posture	7.5	9	8	13.3	7.7
Developmental delay	19.8	10.1	6.5	6.1	6.4
Education	0.9	0	0.5	0	0
Emotional health	0.9	6.7	4.5	8.9	5.1
Health-wellness	3.8	10.7	10.9	8.3	17.3
Injury-surgery-trauma	12.3	13.5	14.4	11.7	8.3
Mobility	10.4	16.9	10	8.3	5.8
Neurological	10.4	12.9	16.4	12.2	16.7
Performing arts	4.7	2.8	3	2.2	1.9
Quality of life-function	8.5	6.2	6.5	7.2	5.1
Sport-athletics	4.7	1.7	8	6.1	9
Other	16	9.6	11.4	15.6	16.7

Numbers and percentages of responders.

^a^Upper quarter includes cervical, thoracic, shoulder girdle and arm combinations.

^b^Lower quarter includes lumbar, pelvic girdle and leg combinations.

Two hundred eighty-eight responders (73.5% of 392) identified the percentages of their students who came from select referral sources. Self-referral was most common (mean = 26.9% ± 28.0%, 95% CI 23.6% to 30.1%), followed by referral from a conventional health care provider (mean = 22.3% ± 29.3%, 95% CI 18.9% to 25.7%). There were 216 responders (55.1% of 392) who reported that students self-referred and provided the percentages of those students who learned about the responders’ practices through 11 sources. The FGNA online directory (mean = 23.0% ± 30.5%, 95% CI 18.9% to 27.1%) was the most frequent specific source of practice information.

Results from 287 responders (73.2% of 392) regarding the percentages of 11 sources of payment for services indicated that 65.2% ± 35.7% (95% CI 61.1% to 69.4%) of students paid out of pocket. For another perspective on payment sources, we grouped 285 responders (72.7% of 392) into 8 certification/credential combinations: 1) *Feldenkrais Method* (FM) only, 2) FM plus conventional health care (CHC), 3) FM plus CAM, 4) FM plus certifications that were not specific (NS) to CHC or CAM, 5) FM plus CHC plus CAM, 6) FM plus CHC plus NS, 7) FM plus CAM plus NS, and 8) FM plus CHC plus CAM plus NS. The 80 responders who were FM plus CHC on average had the highest percentages of payments from Medicaid (mean = 4.9% ± 14.5%), Medicare mean = 15.5% ± 21.9%), private insurance (mean = 22.2% ± 24.2%), and workers compensation (mean = 2.5% ± 7.0%); and the lowest percentage of payment by self-pay (mean = 34.3% ± 35.5%).

We asked responders to review where they provided services (where they gave lessons) and identify the percentages of their practices that occurred in those locations. Of the 288 responders (73.5% of 392), 41.3% ± 42.1% (95% CI 36.4% to 46.2%) practiced in offices located outside their homes, while 28.6% ± 35.6% (95% CI 24.5% to 32.7%) practiced in offices inside their homes.

Feedback from 284 responders (72.4% of 392) indicated the most common of 7 practice settings was a solo practice in a traditional format (mean = 53.8% ± 43.0%, 95% CI 48.8% to 58.8%). By grouping 282 responders (71.9% of 392) into 8 certification combinations, we identified that FM only responders were most likely to have solo traditional settings (mean = 72.5% ± 37.1%) and to teach within *Feldenkrais* Professional Training Programs (mean 3.4%. ± 12.6%). FM plus CHC responders were most likely to work in a multidisciplinary group practice of *Feldenkrais Teachers* plus CHC providers and/or CAM providers (mean 38.7% ± 45.0%).

We asked the 78 responders (19.9% of 392) who reported they practiced within a multidisciplinary group to list up to 4 of the most common types of colleagues or practitioners they worked with other than *Feldenkrais Teachers*. We categorized the types of colleagues into 3 groups: conventional, CAM, and other. The first 2 categories followed the definitions of terms (see Additional file 1) for CHC provider and CAM provider. Across the first to fourth most common types of colleagues, responders were most likely to work with conventional providers (low of 46.9% for fourth to high of 74.4% for first), followed by CAM, and lastly other providers. When we considered common types of colleagues by certification combinations, the responders most likely to be colleagues with conventional providers were those with FM plus CHC (low of 58.6% for first to high of 71.9% for second). FM only responders were most likely to have CAM colleagues (low of 41.2% for first to high of 54.5% for fourth). FM only (first: 66.7% and second: 44.4%) and FM plus CHC (third and fourth: 33.3%) responders were most likely to work with other types of colleagues.

Lastly, we asked responders to review whether or not they combined other approaches in which they were licensed or certified with the *Feldenkrais Method*. Over half of the services offered by 280 responders (71.4% of 392) involved *Feldenkrais Method* lessons alone (mean = 56.1% ± 42.5%, 95% CI 51.1% to 61.1%). The most common combination was with conventional health care (mean = 16.9% ± 33.3%, 95% CI 13.0% to 20.8%).

### Review of representative month during the past 12 months (January - December 2010)

For this section, we asked responders to review their *Feldenkrais Method* practice during the previous 12 months and identify a typical, representative month (average; not the busiest, not the slowest). Responders answered questions similar to those in the prior section. In screening these data, we omitted some responses due to inconsistencies in reporting (i.e., despite instructions, some responders reported percentages instead of numbers). The 224 responders (57.1% of the initial 392) most often chose October (18.8%) as their representative month. They selected July the least (2.2%). The results for the representative month were comparable in all areas to the review of the past 12 months.

### Open comment

In this last section, we gave responders the opportunity to share information about their *Feldenkrais Method* practices that was not addressed elsewhere in the survey. We analyzed comments from 101 responders (25.8% of 392) and identified 5 main themes.

First, as suggested by the earlier comments about integrated practice, several responders thought their skills in another profession were significantly enhanced by their *Feldenkrais* training. Here are 3 example quotations:

• My dance teaching is infused with *Feldenkrais* [*Method*] and it is for this reason I am hired.”

• My skill as a PT [physical therapist] has sky rocketed since my training in the *Feldenkrais Method*. Clients and physicians can tell there is ‘something different’ about how I work. Since the *Feldenkrais* Training, I will never be bored at work now.”

• Even though I had been a social worker for many years, I did not feel capable of being a therapist before I completed my *Feldenkrais* training; it was only after learning to be with people in a Feldy-specific [*sic*] way that I felt I could handle it and had something to offer.”

Second, many statements clustered under the umbrella of practice challenges. Responders wrote about the difficulties of explaining what the *Feldenkrais Method* is and issues common to self-employed practitioners and small businesses (e.g., building a practice, cost of health insurance, impact of the great recession, practicing in smaller communities, marketing, governmental and regulatory constraints, and record keeping). Several described struggling with these challenges, while some highlighted paths of success. These quotations exemplify this theme:

• I would like to have more of my income come from my *Feldenkrais* practice however I find the following to limit my ability to make my *Feldenkrais* practice my sole income provider, or even more of my total income:

very difficult to market *Feldenkrais* [*Method*]

health insurance benefits are difficult for a self-employed person to obtain

the current recession (poor economy) is limiting people from paying for self improvement methods/therapies such as *Feldenkrais* [*Method*].”

• I had a *Feldenkrais* office for a few years but struggled to pay the rent. There was simply not enough business for me in this little town with less than 9000 citizens. I also compete with more and more yoga teachers, massage therapists, and a Rolfer. . . . I find it too cumbersome to always explain in many sentences the difference between *Feldenkrais* [*Method*], yoga and other methods. I decided to let go of any ideas to make money with it.”

• I continually hear that 'we' are hard to find or know about. As a profession we need more mainstream exposure, mainstream magazines, advertising, articles etc.”

• I have opened a business, but the result is Zero, I practice on friends and family at the moment. But to touch someone in CT [Connecticut] you have to be a massage therapist, and now I am a [massage therapy] student . . . and then I can promote the *Feldenkrais* in a lawful way.”

• To be clear, I do not have accurate records of my classes in particular.”

• I teach ATM lessons in rented space. I have changed locations 4 times in the last 3 years and finally found a space that is working.”

• Persistence, stability and good communication skills are essential ingredients for a private practice using the *Feldenkrais Method*.”

Third, responders’ comments demonstrated the variety of views that teachers have about their practice of the *Feldenkrais Method*. This ranged from personal use to a hobby, a retirement activity, a desired part-time practice, a part-time practice seeking growth, to a full-time business. The following 6 quotations illustrate this range of practice.

• . . . My involvement with the *Feldenkrais Method* is basically personal.”

• I am fortunate that I have another source of income that requires very little time. I feel no pressure to build a *Feldenkrais* practice. I tend to treat it as a hobby, and not a business.”

• . . . I don't need the money or the bookkeeping. Also, I travel with my retired husband and am not always available.”

• Dating 10 years from my personal ‘discovery’ of the *Feldenkrais Method* as a student in serious need, I graduated from basic Training with an express intention (call it a business plan..?): I wanted my early retirement from my (previous) career (enjoying gainful employment in sales & marketing) to dovetail with the commencement of a part-time practice as self-employed *Feldenkrais Teacher*. After 2.5 years since graduation, I have steady part-time workload teaching 4–5 ATM classes per week with an occasional FI student or 2. You might say I have taken my bows [*sic*] of poverty and love it! My income from *Feldenkrais* practice covers all expenses associated with the practice (Guild membership, insurance, room rental, advanced training, books & supplies) and provides me flexible spending over and above what I derive from other sources to meet my fixed expenses. I am very grateful that I do not have to depend upon income from a traditional *Feldenkrais* practice to meet all my financial needs.”

• I love the *Feldenkrais Method* and I'm working and ready to increase my practice in a ‘Wellness Center’”.

• I stopped practicing in 2004; during and prior to that, I supported myself and my family on a very full (40 plus hours a week) *Feldenkrais* practice.”

Fourth, several responders made clear that they personally value the *Feldenkrais Method.* Closely related to this value was the desire to share with others what has been so meaningful to them. These 5 quotations point to this theme.

• The method remains a lifeline, an anchor for my curiosity and a sense of joy and calm in my life.”

• In 1976 I met a dancer whose father had helped Moshe [Feldenkrais] come to the US on his first trip. She taught me several ATMs. I was hooked. It was water when I was parched--an answer to a question I had not yet formulated.”

• I feel it is something that I give back for the help that the *Feldenkrais Method* did for me.”

• Since I am retired I do not advertise my practice. . . . I charge a low fee because I do not want money to be the reason someone says 'no' to learning about the Method. I justify all of the above because I have received many personal benefits from *Feldenkrais* [*Method*]. And because of that I want to share it with anyone interested. My aim is not to make money.”

• I see 2 students pro bono on average per month but I do not see that as payment but a mission.”

Finally, despite any challenges in building and maintaining *Feldenkrais* practices, many responders expressed their deep commitment to the value of the *Feldenkrais Method* and to seeing it grow. Here are 3 exemplar quotations.

• I love my work and the knowledge that it really helps to make life better for those I work with!”

• I appreciate anything anyone can do to further the profile of this valuable Method. I would love to do more as a practitioner and have it be more recognized.”

• If what we are doing is to improve one's life we need to let people know it does, the *Feldenkrais Method* has BENEFITS for them, not a lot of community/professional explanations and emphasis on a vague concept of 'learning'. . . . We need a several year plan to expose our work. What happens is we get caught in jargon about descriptions and forget that to sell something you have to make it needed and useful. And we DO have to sell our Method, we have to make it desirable, needed, easy and useful.”

## Discussion

This survey is a significant expansion of a preliminary survey [23] of US *Guild Certified Feldenkrais Teachers*. We invited all teachers to provide information on many more topics. Despite the sizeable increase, overall response rates were similar: 32.3% in 2010, and 30.5% here. In this section, we reflect on the current results, use them to address several questions raised by the preliminary survey, situate these results with other CAM studies, and suggest areas in need of more inquiry.

The number of *Feldenkrais Teachers*, although small compared to other CAM disciplines such as massage therapy [25], increased dramatically in the decades after Feldenkrais’ death in 1984. Nearly 90% of responders completed their training between 1991 and 2010. During that time period, CAM use in general increased in the US to about 40% among adults [1,29] and 11% among children [1].

While the increase in *Feldenkrais Teachers* was stable in the past 2 decades, the numbers of teachers rising to the ranks of Assistant Trainer and Trainer were lower in the 2000s than in the 1990s. If the profession does not address this pattern, it may reduce the capacity for training *Feldenkrais Teachers* moving forward, given the average ages of 57.9 for Assistant Trainers and 62.2 for Trainers.

*Feldenkrais Teachers* were typically well educated in a wide range of fields. Ninety percent held bachelor’s degrees or higher. Physical therapy was the most frequent area of study. As was the case in the preliminary survey, physical therapist was the most commonly held conventional health care credential and massage therapist was the most common CAM credential. Percentages were similar in both surveys. Sixty percent of respondents in the current survey held credentials in conventional health care, CAM, or another discipline outside of health care. Unlike in the preliminary survey, we could identify here that most conventional health care providers held 1 such license. Conversely, over half of CAM providers held more than 1 CAM credential. It may be more prevalent for CAM practitioners to hold more than 1 credential. For example, studies reported half of Australian naturopaths [30] held credentials in other CAM fields, while up to a third of acupuncturists, chiropractors, massage therapists, and naturopaths were licensed in another CAM or conventional health care field [25].

Overall, our results suggest that *Feldenkrais Teachers* have educational backgrounds that complement and enhance their *Feldenkrais* certifications. Open comments indicate the converse is also true, as many responders stated that the addition of *Feldenkrais* certification significantly improved their abilities in health care and related disciplines.

Responders used a broad range of tools to promote their practices. The most common were the FGNA print directory and online directory. As was the case for patients of naturopaths, acupuncturists, massage therapists and chiropractors [26], students most often self-referred to *Feldenkrais Teachers*. Students frequently found their teachers through the online directory. Given that only members of FGNA have access to listings in the online directory, membership in FGNA can be an important investment in practice promotion for *Feldenkrais Teachers*. This resource can also serve as a tool for health care professionals who wish to refer patients for complementary services or follow-up care.

Over 80% of *Feldenkrais Teachers* were women. We did not locate other gender data for *Feldenkrais Teachers*, but did find studies that identified greater proportions of women acupuncturists [25,30], herbalists [30], massage therapists [25,31], and naturopaths [24,25,30]. *Feldenkrais Teachers* overall were somewhat older than other CAM practitioners. Over 75% of *Feldenkrais Teachers* were aged 50 or older. Studies of other CAM providers reported mean ages in the 40s [25,31]. Comparable to these CAM providers, with the exception of acupuncturists [25,31], *Feldenkrais Teachers* were majority white and not Hispanic, Latino or Spanish.

Similarly, the students of *Feldenkrais Teachers* were mostly women and mostly 45–64 years old. Other CAM studies also identified that women were more frequent consumers [1,24,26]. Barnes [1] found users of manipulative and body-based practices, the category that includes the *Feldenkrais Method*, were mostly 30–59 years old. Cherkin [26] used wider age ranges and reported most patients of naturopaths, acupuncturists, massage therapists and chiropractors were 15–64 years old.

For both the preliminary survey and the current study, most *Feldenkrais Teachers* practiced in California and New York. Regional rankings varied somewhat between surveys. Responders to the first survey were mostly located in the West, followed by the Northeast, South and Midwest [23]. The Northeast and South reversed rankings in the current survey. Both distributions differ from the 2010 US Census data that identified the Midwest as most populous region, followed by the Northeast, West, and South [32]. From the perspective of utilization of manipulative and body-based practices, Barnes [1] reported highest usage by adults in the West, then Midwest, Northeast and South. For children, West and Midwest were similar, followed by Northeast and South. Another factor to consider in understanding the distribution of *Feldenkrais Teachers* is the location of *Feldenkrais* Professional Training Programs. The first occurred in California and the second in Massachusetts. Subsequently, nearly 40% of responders graduated from trainings held in California, 6.9% in Illinois, and 6.6% in New York. The distribution of *Feldenkrais Teachers* may reflect an interaction among training history and population-based factors.

Similar percentages of men and women responders had individual gross incomes above and below $50,000. Thus, half or more had incomes above the US medians for full-time workers in 2010 (men $47,715, women $36,931) [33]. Higher percentages of women than men had incomes below $25,000 and between $50,000 and $75,000, while a higher percentage of men had incomes above $75,000. In comparison, Whalen [34] reported that men who were chiropractors earned 49% more than women. Hale’s survey [30] of acupuncturists, herbalists and naturopaths found men were more likely than women to earn 81%-100% of their income from their practices. Women were more likely to earn less than $30,000 (Australian) while men were more likely to earn over $100,000. Thus, while gender differences existed in earnings among US workers and CAM practitioners, *Feldenkrais Teachers* were likely to earn more than the median income with under half of their annual income coming from their *Feldenkrais* practices.

The finding that just over half of responders did not identify *Feldenkrais Teacher* as their primary occupation likely influenced the percentage of income from *Feldenkrais Method* practice. This finding may also be reflected in our results that suggest *Feldenkrais Teachers* on average had part-time practices. Results from the preliminary survey were similar with means of 7.6 students per week for individual lessons, 8.4 students per week for group lessons, and 2.9 new students per month [23]. Reports for massage therapists ranged from 10 to 19.5 visits per week [25,28,31]. Conversely, acupuncturists and naturopaths averaged about 30 visits a week, meaning most had full-time practices [25]. If desired, many *Feldenkrais Teachers* have room to grow their practices and their incomes.

Students of *Feldenkrais Teachers* were of all ages. The most frequent age group was 45–64 years. This is comparable to the most common age group for all CAM consumers (30–69 years) and manipulative and body-based therapies specifically (30–59 years) [1]. It also nests within the wider age range of most prevalent consumers of services by naturopaths, acupuncturists, massage therapists and chiropractors [26]. The proportion of students who were women was about twice that for men. This gender distribution was similar to other CAM studies that reported higher usage by women [1,24,26].

Our responders reported that pain was the leading reason that students sought their services. Within the pain group, the primary location of pain was the back. Pain [35] and specifically back pain [1,26,29] were top reasons for seeking CAM services in other reports. Subsequently, reasons cited for seeking lessons focused increasingly on non-pain concerns. More commonly reported reasons included neurological conditions, recovery from injury-surgery-trauma, health-wellness, mobility, and balance-posture. This pattern is similar to that reported in a study of CAM providers that included *Feldenkrais Teachers*[35]. Although there is very little evidence of adverse effects from *Feldenkrais* lessons [3], research should examine the safety of this somatic learning method.

Research supporting the effectiveness of the *Feldenkrais Method* for the cited reasons is broad yet relatively limited compared to more widely used interventions [3]. However, there are multiple studies supporting effectiveness for people who come for *Feldenkrais* lessons to improve pain [11,36,37] and particularly back pain [13,14], and improve balance [8-10]. Given that 1) *Feldenkrais* students were mostly 45 years and older, 2) people in this age range account for a disproportionate amount of health care costs [2] and 3) falls in seniors are a serious contributor to nonfatal and fatal injuries [38], *Feldenkrais Teachers* may be well positioned to offer effective health promotion interventions to impact back pain and enhance balance. Appropriately designed studies could determine if *Feldenkrais* lessons that improve balance in turn reduce the risk of falls.

While a quarter of students self-referred to *Feldenkrais Teachers* during 2010, conventional health care providers referred over 20% of the students to *Feldenkrais* practices. The implication is that a substantial number of students were seeing both conventional health care providers and *Feldenkrais Teachers*. This is consistent with other studies that reported health care consumers often combine conventional health care with CAM practices [1,29,39-41]. However, it is not always clear whether conventional health care providers are aware of their patients’ use of CAM practices. A survey of patients with arthritis reported that most used at least 1 type of CAM and a smaller majority had discussed this with their physicians [42]. A study of rehabilitation medicine physicians indicated most had been asked about CAM by their patients and most had spoken against some form of CAM use [40]. Broader surveys reported that less than half of patients who combined CAM and physician visits discussed the CAM visits with their physicians [29]. Reasons for this communication gap included the failure of physicians to ask patients about CAM use and perceptions of patients that physicians would be judgemental [43]. Patients did not expect physicians to be experts in CAM, but desired open-minded inquiries from their physicians [43]. In another study, many patients indicated they would prefer providers who combine CAM and conventional health care [44]. This may be a factor favoring the practices of *Feldenkrais Teachers* who also hold conventional health care licenses. Future studies could inquire directly of *Feldenkrais* students about their usage of CAM and conventional health care providers, and their communication with their providers.

The findings of the present study offer much more detailed information about the characteristics and practice profiles of *Feldenkrais Teachers* to the broader health care community. This information, combined with existing and subsequent research, can enhance communication among all members of a person’s health care team. Opportunities for collaboration and continuing education may emerge that mutually strengthen providers’ practices and benefit patient-centered care.

Self-pay was the most prevalent form of payment for *Feldenkrais* services. This is consistent with several studies of CAM practices and usage that reported consumers often pay for services directly [24,26,29,31,34,41]. Some CAM practitioners (e.g., naturopaths and acupuncturists) were more likely to be included in insurance plans and therefore had notable payments from such sources [26]. Among *Feldenkrais Teachers*, those who held a conventional health care credential were more likely to receive payment from insurers and less likely to receive direct pay from students. While individual FI lessons allow *Feldenkrais Teachers* to customize lessons to students, group ATM lessons provide low cost access for those with limited financial resources.

*Feldenkrais Teachers* were most likely to see students for lessons in offices outside their homes, with offices in their homes being the next most common location. Hale [30] reported a similar location pattern for Australian CAM practitioners. Overall, *Feldenkrais Teachers* were most likely to have solo practices, especially if they did not hold other credentials. In contrast, *Feldenkrais Teachers* who were also conventional health care providers were most likely to have multidisciplinary group practices that most likely included other conventional health care providers. Acupuncturists, chiropractors, massage therapists and naturopaths also commonly had solo practices [25]. Their multi-professional group practices included CAM and conventional health care providers [25].

Similar to the preliminary survey [23], over half of the present responders offered purely, exclusively *Feldenkrais Method* lessons. A distant second was *Feldenkrais Method* and conventional health care. While we know that other CAM providers have credentials in other health professions [25], it is not clear how they combine them in their practices.

Whether expanding on teaching the *Feldenkrais Method* in integrated formats or offering open comments about their practices, *Feldenkrais Teachers* expressed perceptions of the value that the *Feldenkrais Method* added to their personal and professional lives. In spite of the typical challenges of self-employment and small business management, and the particular challenge of marketing a distinctive somatic approach to learning, *Feldenkrais Teachers* stated their convictions in the method. The challenge of succinctly describing the *Feldenkrais Method* and positioning it relative to more popular categories was mirrored by the myriad ways that *Feldenkrais Teachers* put their professional training into practice. The complexity of the method, while making a short description difficult, apparently facilitated its application to many situations for both teachers and students.

The results of this study have expanded knowledge about the characteristics and practice patterns of *Feldenkrais Teachers*. These findings are consistent with anecdotal accounts about the diverse backgrounds of teachers and the wide range of reasons students seek their services. Still, there is considerable room for more research. We acknowledge the present limitation that this is a retrospective study and subject to recall bias. We instructed responders to review their records as they completed the survey, but accept that may not have occurred in all instances. Additionally, some responders commented that the survey helped them recognize areas for improved record keeping. Prospective inquiry could reduce or eliminate many of these concerns. The details of utilization of *Feldenkrais* services by students remain unclear. The number of individual lessons or group lessons a student attends over time is not known from our results. This may vary with the reasons students seek the services of *Feldenkrais Teachers*, for example, back pain vs. wellness. Future studies could query students directly for information about why they sought *Feldenkrais* lessons and what other providers they were using to assist with their health and wellness.

The present study grew from a preliminary study and offered participation to the census of US *Feldenkrais Teachers* via web-based or paper formats. While training standards are similar worldwide, findings are limited to the US and might differ from surveys of teachers in other countries. Strengths of the survey include its incorporation of questions adapted from prior surveys of CAM providers and consumers, inclusion of items specific to the *Feldenkrais Method*, and rounds of review by focus groups of *Feldenkrais Teachers* before finalization. No response bias existed based on geographical distribution or gender. However, Assistant Trainers and Trainers were more likely to respond than teachers without these advanced credentials. The main limitation is the overall response rate of 30.5%; this may indicate a risk of lower accuracy for the survey results. However, responses for questions in the preliminary survey that parallel ones in the present survey provided comparable results. As a check on the reliability of the 12-month data, we asked respondents to provide information for representative months of practice for comparison. Responses for the representative month review and the 12-month review were similar. While these factors provide support for the reliability and validity of this survey, we cannot eliminate the possibility of non-response bias in our results.

## Conclusions

The results of this survey of US *Guild Certified Feldenkrais Teachers* indicated that 90% were college educated in a broad range of disciplines. The most common was physical therapy. Over half held 1 or more credentials in conventional health care, CAM, or disciplines outside of health care. The most common in each category were physical therapist, massage therapist, and education. The growth in the number of *Feldenkrais Teachers* was significant during the 1990s and 2000s and occurred at a faster rate than that for Certified *Feldenkrais* Assistant Trainers and Certified *Feldenkrais* Trainers. *Feldenkrais Teachers* were predominantly located in the states of California and New York, and in the West region. Most were women and ages 50 years or older. Practices varied in size and settings. Just under half of *Feldenkrais Teachers* earned 20% or less of their gross income from their practices, while a quarter earned over 80% of their income from *Feldenkrais* practice. Information about visits and income indicate most *Feldenkrais Teachers* had part-time practices. *Feldenkrais Teachers* mostly had offices outside of their homes, operated solo practices, offered purely *Feldenkrais Method* lessons, and were directly paid by students. Those who were also conventional health care providers were likely to have multidisciplinary group practices with other conventional health care providers, combine *Feldenkrais Method* with their licensed practice, and be paid more often by insurers. Students were mostly women and mostly 45–64 years old. Their primary reason for seeking the services of *Feldenkrais Teachers* was to help with pain, followed by non-pain reasons. A quarter of students self-referred while a fifth was referred by conventional health care providers to *Feldenkrais Teachers*. Consistent with the description of the *Feldenkrais Method* as a learning approach, teachers and students utilized the *Feldenkrais Method* in a wide and diverse array of settings and applications.

These findings can assist decision-making by stakeholders in conventional and CAM health care in several ways. First, it may promote strategic planning and practice development for *Feldenkrais Teachers* through appropriate access to certification, and enhanced marketing strategies and practice management. Second, this study can improve communication among *Feldenkrais Teachers*, health care consumers and providers that informs decision-making and fosters mutually beneficial alliances. Finally, researchers can use the results to design studies of the characteristics of *Feldenkrais* students and investigations of the safety and effectiveness of the *Feldenkrais Method*.

## Competing interests

The first author is a *Guild Certified Feldenkrais Teacher* and member of the *Feldenkrais Guild* of North America (FGNA). She has received small honoraria from FGNA for presenting workshops at its 2005 and 2009 annual conferences.

## Authors’ contributions

PB conceived of the study and coordinated and participated in all aspects of its conduct. NN contributed to analysis and interpretation of data, and helped to draft the manuscript. SG contributed to survey development, performed statistical analyses, and helped to draft the manuscript. All authors read and approved the final manuscript.

## Pre-publication history

The pre-publication history for this paper can be accessed here:

http://www.biomedcentral.com/1472-6882/14/217/prepub

## Supplementary Material

Additional file 1 Definitions of terms provided to teachers in survey introduction. Click here for file
