# Supplementary material for: United States Guild Certified Feldenkrais Teachers®: a survey of characteristics and practice patterns
Source: BMC Complement Altern Med. 2014 Jul 2;14:217. doi: 10.1186/1472-6882-14-217 (PMC4086994; doi:10.1186/1472-6882-14-217)
Supplement: Additional file 1 — Definitions of terms provided to teachers in survey introduction. [file 1472-6882-14-217-S1.pdf]

Additional file 1 – Definitions of terms provided to teachers in survey introduction

Please read these definitions of **bolded terms** before you begin the survey. Keep these definitions in mind as you provide your responses.

Guild Certified Feldenkrais Practitioner*^{CM}** (GCFP): a graduate of an accredited Feldenkrais® Professional Training Program who is currently certified by the *Feldenkrais Guild*® of North America (FGNA). Some prefer the term ***Guild Certified Feldenkrais Teacher®.

Guild Certified Feldenkrais Teacher (GCFT): a graduate of an accredited Feldenkrais Professional Training Program who is currently certified by the *Feldenkrais Guild* of North America (FGNA). Some prefer the term *Guild Certified Feldenkrais Practitioner*.

Feldenkrais* practitioners**: in this survey, this term refers to both ***Guild Certified Feldenkrais Practitioners and ***Guild Certified Feldenkrais Teachers***.

Certified *Feldenkrais* Assistant Trainer: in addition to being a ***Feldenkrais* practitioner**, this person is recognized by the North American Training Accreditation Board as qualified to assist in the instruction of students within *Feldenkrais* Professional Training Programs.

Certified *Feldenkrais* Trainer: in addition to being a ***Feldenkrais* practitioner**, this person is recognized by the North American Training Accreditation Board as qualified to instruct students within *Feldenkrais* Professional Training Programs and serve as educational director of those programs.

Techniques based on the teachings of Dr. Feldenkrais: these are techniques that have been developed by ***Feldenkrais* practitioners**. They have separate names and certification standards from the *Feldenkrais Method*. They include:

- Anat Baniel Method^{SM}
- Bones for Life®
- Child'Space - Chava Shelhav Method
- Core Integration (DellaGrotte)
- Sounder Sleep System®

Traditional health care provider: typically, these are licensed or certified practitioners in health care fields that are recognized by the American Medical Association and similar groups operating within a conventional, Western medical model. They include:

- Art/recreation therapist
- Athletic trainer, certified
- Audiologist
- Chiropractor
- Clinical psychologist
- Dentist
- Dietician
- Licensed practical or vocational nurse
- Marriage and family therapist

- Mental health counselor
- Nurse and other advanced nursing
- Nurses aide
- Nutritionist
- Occupational therapist assistant
- Occupational therapist
- Optometrist
- Osteopath/doctor of osteopathy
- Physical therapist assistant
- Physical therapist
- Physician assistant
- Physician/medical doctor
- Podiatrist
- Professional counselor, alcohol or family/marriage or substance abuse
- Psychologist
- Rehabilitation therapist
- Respiratory therapist
- School psychologist
- Social worker
- Speech/language pathologist

Complementary and Alternative Medicine (CAM) provider: the National Center for Complementary and Alternative Medicine (NCCAM), a part of the National Institutes of Health, "defines CAM as a group of diverse medical and health care systems, practices, and products that are not generally considered part of conventional medicine." Providers are typically licensed or certified. NCCAM considers the *Feldenkrais Method* to be a CAM practice. Other CAM practices include:

- Acupuncture/traditional Chinese medicine
- Alexander technique
- Ayurveda
- Biofeedback
- Bowen technique
- Healing touch
- Homeopathy
- Johrei
- Manipulation, chiropractic or osteopathic
- Massage therapist
- Meditation
- Naturopathy
- Pilates
- Polarity therapy
- Qi gong
- Reflexology
- Reiki

- Rolfing
- Tai chi
- Therapeutic touch
- Trager bodywork
- Tui Na
- Vortex healing
- Yoga

Primary occupation: this is a person's major form of employment and/or means of earning individual income. This may or may not be one's practice of the *Feldenkrais Method*.

Your practice of the *Feldenkrais Method*: this is how you are applying the method in your work. This may be in a **traditional format** and/or an **integrated format**. Unless otherwise explicitly specified, this includes your use of techniques based on the work of Dr. Feldenkrais in which you are also certified.

Traditional format: refers to a style of practicing the *Feldenkrais Method* in which appointments for individual or group lessons are typically scheduled and often last about an hour. Besides *Functional Integration*® (FI) and *Awareness Through Movement*® (ATM) lessons, the traditional format may also include **workshops** that last a few hours to a few days. The *Feldenkrais Method* is the primary learning method/focus in this format. This format may be common for many practitioners, including those who practice in health care provider settings.

Integrated format: refers to a style of practicing the *Feldenkrais Method* in combination with other occupations or professional contexts in which the *Feldenkrais Method* is a component of interaction, but not the primary learning method/focus. Appointments or schedules may or may not be used, but the *Feldenkrais Method* is not the main approach used throughout the sessions. This format may be used in traditional education settings and many other work settings.

Students: ***Feldenkrais* practitioners** refer to the people they work with in different ways. Terms can vary with practice settings and philosophies. Some common terms include clients, **students**, and patients. Throughout this study, we will use students to refer to the people who seek the services of ***Feldenkrais* practitioners**.

Workshop: this is a way of offering the *Feldenkrais Method* to groups. Sessions are usually longer than typical ATM sessions, are often thematic, and may span more than one date. Workshops may target the public, including people who are not ***Feldenkrais* practitioners**, or may target other ***Feldenkrais* practitioners**. For this survey, a workshop falls within a **traditional format** and has the *Feldenkrais Method* as the primary learning method/focus.
